# Efficacy and effectiveness of anti-VEGF or steroids monotherapy versus combination treatment for macular edema secondary to retinal vein occlusion: a systematic review and meta-analysis

**DOI:** 10.1186/s12886-022-02682-7

**Published:** 2022-12-06

**Authors:** Wuyue Zhang, Yuan Liu, Aimin Sang

**Affiliations:** Eye Institute, Affiliated Hospital of Nantong University, Medical School of Nantong University, Nantong, China

**Keywords:** Retinal vein occlusion, Macular edema, Anti-VEGF, Steroids, Combination therapy

## Abstract

**Background:**

Retinal vein occlusion (RVO) is the main cause of retinal vascular blindness. Laser photocoagulation therapy is the regarded as the standard treatment for branch retinal vein occlusion (BRVO) in the guidelines, but it is not effective for macular edema (ME) secondary to central retinal vein occlusion (CRVO). As anti-VEGF (vascular endothelial growth factor) or steroids monotherapy has been used to treat RVO, but each has its advantages and disadvantages. Our purpose was to evaluate the efficacy and safety of intraocular injection of anti-VEGF combined with steroids versus anti-VEGF or steroids monotherapy for ME secondary to RVO.

**Methods:**

We systematically searched trials on Pubmed, Embase, Cochrane Library, Web of Science and China National Knowledge Infrastructure (CNKI) for RCTs (random clinical trials) or non-RCTs, comparing anti-VEGF or steroids monotherapy to their combination. The primary outcomes were changes in best-corrected visual acuity (BCVA), central macular thickness (CMT) and intraocular pressure (IOP). The pooled data was analyzed by random effects model.

**Findings:**

A total of 10 studies selected from 366 studies were included in this meta-analysis. Our results favored anti-VEGF with steroids combination therapy in comparison with anti-VEGF {pooled SMD (standardized mean difference), 95% CI, -0.16 [-0.28, -0.04], *P* = 0.01} or steroids (pooled SMD, 95% CI, -0.56 [-0.73, -0.40], *P* < 0.00001) alone on changes of BCVA. Compared with anti-VEGF monotherapy group, the combination therapy also had a better effect {pooled MD (mean difference), 95% CI, -9.62 [-17.31, -1.93], *P* = 0.01)} at improvements on CMT. On the changes of IOP, assessment favored that combination therapy was associated with a better relief of IOP compared to steroids monotherapy group (pooled MD, 95% CI, -5.93 [-7.87, -3.99],*P* < 0.00001). What’s more, the incidence of ocular hypertension was lower in the combined treatment group compared with control group treated with steroids alone (Odds Ratio, 95% CI, 0.21 [0.06, 0.77], *P* = 0.02). Results also showed that the combination group can prolong the average time to first anti-VEGF reinjection (MD, 95% CI, 1.74 [0.57, 2.90], *P* = 0.003) compared to control group treated with anti-VEGF alone.

**Conclusion:**

Anti-VEGF with steroids combination treatment can enable a better achievement of improving BCVA, CMT, reducing the risk of increased IOP and improving patient prognosis compared to anti-VEGF or steroids therapy alone, lengthening the average time to anti-VEGF reinjection with reducing the injections during follow-up.

**Supplementary Information:**

The online version contains supplementary material available at 10.1186/s12886-022-02682-7.

## Introduction

Retinal vein occlusion (RVO) is the second most common retinal vascular disease after diabetic retinopathy, leading cause of retinal vascular blindness. It can be classified into branch retinal vein occlusion (BRVO) and central retinal vein occlusion (CRVO) according to the location of vascular occlusion [1]. Macular edema (ME) is the major complication of retinal vein occlusion and severely affects patients' vision [2]. Laser photocoagulation has been the standard treatment for ME secondary to branch retinal vein occlusion (BRVO). Furthermore, although laser therapy is the standard treatment for patients with ME secondary to BRVO, it was not beneficial for ME secondary to CRVO [3].

The occurrence and development of RVO is caused by multiple whole and local pathogenic factors. Current researches show that cytokines are involved in the pathophysiology in RVO. Noma et al. [4] have confirmed that the levels of vascular endothelial growthfactor (VEGF) and VEGF receptor (VEGF-R) is increasing in aqueous humor of patients with RVO. The increase of VEGF-R concentration in the ischemic RVO group was more significant than that in the non-ischemic RVO group, and was positively correlated with the degree of ME.

According to the European Society of Retina Specialists (EURETINA), vitreous injection of anti-VEGF drugs is currently the primary treatment for RVO secondary to ME, no matter it is CRVO or BRVO. Once ME is found, vitreous injection should be carried out in time. Continuous injection should be at least 3 months, once a month, until the vision acuity has been stable, debating whether 3 + PRN or 6 + PRN should be chosen for application [5]. Due to the short half-life of anti-VEGF drugs and the continuous release of VEGF in the eye, however, repeated injections are often needed to maintain the intraocular drug concentration to achieve the purpose of treating ME, which is expensive and not easily accepted by patients.

Inflammatory processes has been considered to be crucial in the pathogenesis of ME [6]. Steroids, such as triamcinolone acetonide (TA) and dexamethasone vitreous implant (DEX), can offer a significant non-specific anti-inflammatory efficacy and a significant clinical effect on ME secondary to RVO. TA, which is difficult to dissolve in water and is hard to be absorbed, has a long-acting glucocorticoid with anti-inflammatory and vasoconstrictive effect. The effective drug duration can be up to 2–3 weeks after intravitreal injection of triamcinolone acetonide (IVTA). The drug can cross the blood-retina barrier, act on the surface of the retina directly, inhibit the generation and release of inflammatory factors, alleviate vascular inflammation, reduce vascular permeability, down-regulates VEGF and stabilise the blood-retina barrier, so as to achieve the purpose of treating ME. DEX is a biodegradable sustained-release implant containing 0.7 mg dexamethasone. The concentration reaches a peak at 2 months after implantation in vitreous cavity, followed by sustainable release of dexamethasone for up to 6 months. Compared with TA, dexamethasone sustained-release implant (Ozurdex) offers a stronger and longer effect, maintaining the intraocular drug concentration more stably [7].

Single or repeated injection of TA or Ozurdex can significantly improve the visual acuity of patients with RVO, especially in the treatment of recurrent RVO macular edema [8], but it may cause increased intraocular pressure, cataract and other common side effects of glucocorticoid drugs, which deserves attention and vigilance [9].

Therefore, intravitreal injection of anti-VEGF drugs is a first-line approach for ME secondary to RVO, and steroid sustained-release drugs also represent an effective alternative for ME in RVO [10]. Combination therapy of anti-VEGF and steroids, overcoming the disadvantage of both monotherapies, may be considered as a potential option for ME patients who do not respond to either anti-VEGF or steroids alone.

In this systematic review and meta-analysis, we aimed to explore the efficacy and safety of anti-VEGF or steroids monotherapy vs. combination treatment of anti-VEGF plus steroids for ME secondary to RVO, and to verify the differences in the effects of treatment approaches on BRVO and CRVO, so as to provide clinical reference for the treatment of RVO.

## Materials and methods

### Search strategy and selection criteria

This meta-analysis is reported in accordance with the Systematic Reviews and Meta-Analyses (PRISMA) Statement. The registration number in PROSPERO is CRD42022332751.

Trials were identified by a comprehensive search on Pubmed, Embase, Cochrane Library, Web of Science and CNKI. For studies published in Chinese journals, only journals indexed by The key magazine of China technology were considered to reduce publication bias. Trials on the ClinicalTrials.gov website, including those had been terminated, were excluded. We selected studies without date limits and language restrictions. The main search strategy were as follows: #1: vascular endothelial growth factor OR VEGF OR bevacizumab OR ranibizumab OR aflibercept OR pegaptanib OR avastin OR conbercept; #2: glucocorticoid OR steroids OR Ozurdex OR triamcinolone acetonide OR dexamethasone; #3: retinal vein occlusion; #4: Macular edema; #5: Controlled Trial; #6: #1 AND #2 AND #3 AND #4 AND #5.

### Study selection and data extraction

We considered studies as eligible for inclusion if they were clinical trials including randomized and non-randomized controlled trials, comparing control group as monotherapy of steroids or anti-VEGF treatment with experimental group as a combination of both, including patients with ME secondary to RVO. We recorded the diverse types of RVO in the included cases, but we didn't make that distinction when searching. Trials that included laser therapy in therapeutic scheme and provided data lacking specific mean or standard deviation (SD), as well as non-clinical trials, were excluded.

The outcomes we assessed included changes on BCVA and changes on CMT, as well as side effects such as IOP, cataract, macular epiretinal membrane formation, endophthalmitis, retinal detachment, vitreous hemorrhage and glaucoma. Two investigators (WY Zhang, Aimin Sang) independently screened all trials retrieved by search strategy described above, with one investigator reviewing the titles and abstracts and the other assessing the full texts without disagreement.

The data of included studies that we extracted or calculated were as follows: study ID (first author), year, design, sample size (participants), male/female, age, etiological factor, case/control (monotherapy/combination therapy), details of injection, follow-up periods, selection criteria and evaluating parameters including changes in BCVA (mean [SD]), changes in CMT (mean [SD]), changes in IOP (mean [SD]).

Due to the different study types of RCTs and non-RCTs, we respectively adopted different methods for risk of bias assessment. Two independent reviewers (WY Zhang, Aimin Sang) assessed the risk of bias of RCTs based on PRISMA recommendations. The risk of bias of non-RCTs (non-random clinical trials) was assessed according to “Methodological Index for Non-randomized Studies” (MINORS) [11], according to which the items are scored 0 (not reported), 1 (reported but inadequate) or 2 (reported and adequate). The global ideal score being 16 for non-comparative studies and 24 for comparative studies). A total of 12 characteristics of non-RCTS were evaluated in our study.

### Statistical analysis

We evaluated the efficacy of anti-VEGF or steroids monotherapy and combination treatment on changes in BCVA, central choroidal thickness and IOP to assess the improvement effect for ME secondary to RVO with a time-dependent subgroup analysis designed according to different follow-up durations. We analyzed outcomes as continuous variables and reported absolute differences in arithmetic mean before and after intervention, using the mean difference (MD) with 95% confidence interval (CI). Continuous data were provided as mean and SD. We used odds ratio (OR) with 95% CI in the assessment of adverse events such as cataracts and ocular hypertension. All the data synthesis were based on random effects models. We used the Cochran Q test to evaluate the magnitude of heterogeneity among the included studies. When the value of p was less than 0.1, we regarded it as evidence of heterogeneity. I^2^ testing was also performed to assess the heterogeneity, with values greater than 50% considered to indicate moderate to high heterogeneity. There was moderate heterogeneity between the studies of a few subgroups. Due to the small sample content of each of them, we conducted a sensitivity analysis through leave-one-out method and found that these individual results were consistent with the conclusion of meta-analysis. In addition, We constructed a funnel diagram and used the Begg-Mazumdar’s rank test and the Egger’s regression test to assess publication bias, defining significant publication bias as a *p* value < 0.05. Since the numbers of studies included in other subgroups were less than 5, we only assessed the publication bias of the effect of anti-VEGF monotherapy vs combined therapy on BCVA at 6-month follow-up. The *P* value was less than 0.05, indicating that no significant publication bias was detected (Supplement Table 1, supplement Fig. 1).

**Fig. 1:**
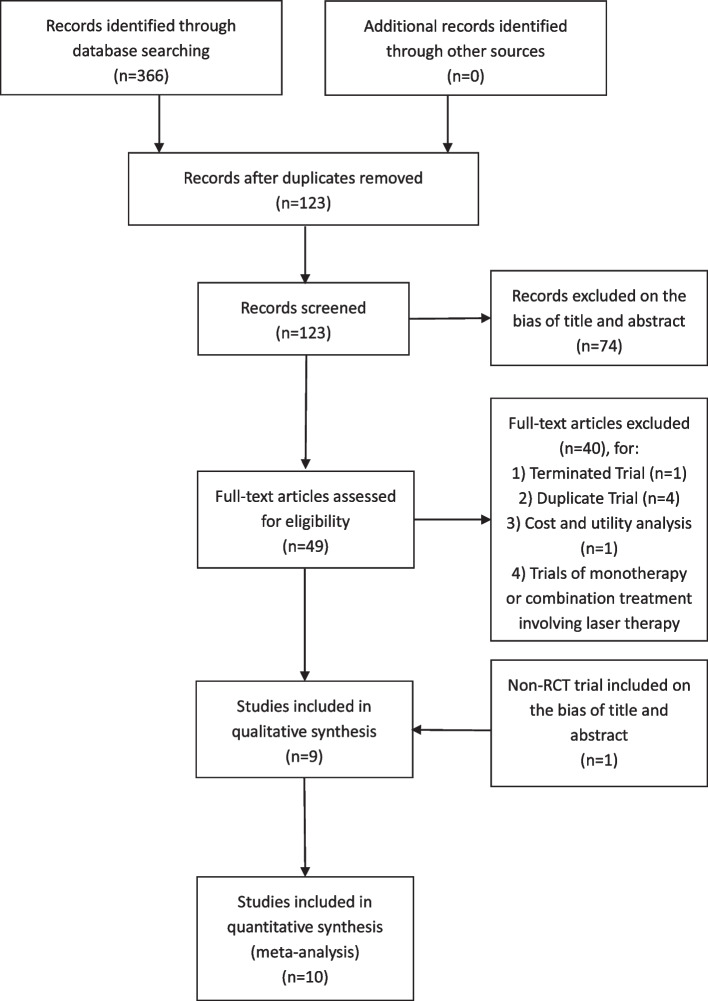
PRISMA Flow diagram of study

We utilized Review Manager (version 5.4, Cochrane Collaboration) and STATA (version 15.1, STATA Corp) for all statistical analyses.

## Results

### Study characteristics

A total of 366 records were identified, of which 10 studies were remained in our analysis after screening (Fig. 1). The 10 studies consisted of 9 RCTs and 1 non-RCT, all published bewteen 2010 and 2022. The main characteristics of all included studies have been shown in Tables 1 and 2. The follow-up period ranged from 1 week to 24 months. Eight trials [12–19] compared the efficacy of anti-VEGF monotherapy and combination therapy for ME secondary to RVO, while four trials [12, 17, 20, 21] compared the efficacy of steroids monotherapy and combination therapy. The control group received sham injection in addition to the monotherapy.

**Table 1:** Characteristics of 9 RCTs and 1 non-RCT, including Study ID, Year, Design, Sample size, Male/Female, Age, Etiological Factor

Study ID(first author)	Year	Design	Sample size(participants)	Male/Female	Age	Etiological Factor
Osman Çekiç [12]	2010	RCT	52	29/23	66.5 ± 0.3; 60.1 ± 3.9; 62.4 ± 1.5	BRVO
Alex S. Willoughby [13]	2018	RCT	38	20/18	66(range 37-)	RVO
J Moon [14]	2016	RCT	41	22/19	60.57 ± 10.68; 58.83 ± 15.66	BRVO
Chuanfeng Fan [15]	2014	2014	57	28/29	range 40–73	CRVO
Peter A. Campochiaro [16]	2017	RCT	46	23/23	68(range 37–91)	BRVO(19)/HRVO(1)/CRVO(26)
Kyungmin Lee [17]	2013	non-RCT	151	16/15(IVTA); 51/44(IVB); 14/11(IVTA + IVB)	57 ± 10(IVTA);58 ± 11(IVB);58 ± 11(IVTA + IVB)	BRVO
Raj K Maturi [18]	2014	RCT	30	10/5(IVB);8/7(IVB + DEX)	67 ± 13(IVB): 69 ± 19(IVB + DEX)	CRVO(15)/BRVO(15)
ZHAO Xue-zhang [20]	2022	RCT	40	17/23	55.35 ± 10.24(IVTA); 56.24 ± 10.03(IVTA + IVC)	CRVO
DUAN Yu-ping [21]	2019	RCT	300	83/67	57.6 ± 5.4(IVTA); 57.5 ± 5.3(IVTA + IVC)	RVO
Zheng Tan [19]	2022	RCT	66	35/31	59.85 ± 8.20(IVA); 60.45 ± 8.00(IVA + IVTA)	CRVO

**Table 2:** Characteristics of 9 RCTs and 1 non-RCT, including Case/Control, Details of Injection, Evaluating Parameters, Follow-up Periods, Selection Criteria

Study ID(first author)	Case/Control(monotherapy/combination therapy)	Details of Injection	Evaluating Parameters	Follow-up Periods	Selection Criteria
Osman Çekiç [12]	17(IVTA)&14(IVB)/ 21(IVTA + IVB)	IVTA: triamcinolone acetonide 4 mg; IVB: bevacizumab 1.25 mg; IVTA + IVB: triamcinolone2mg + bevacizumab 1.25 mg	1) Snellen Visual Acuity; 2) CMT	Mon 1, 3, 6	1) visual acuity of 20/40 or worse; 2) central macular thickness of 250 μm or greater
Alex S. Willoughby [13]	17(IVA)/21(IVA + IVTA)	IVA: Aflibercept 2 mg + Placebo; IVA + IVTA: Aflibercept 2 mg + triamcinolone acetonide 4 mg(Suprachoroidal Injection)	1) vascular choroidal thickness (VCT); 2) stromal choroidal thickness (SCT); 3) total choroidal thickness (TCT); 4) suprachoroidal space (SCS)	Mon 1, 2, 3	1) macular edema due to RVO of ≤ 12 months Duration; 2) best-corrected visual acuity (BCVA) of 20 to 70 ETDRS letters; 3) central subfield thickness (CST) > 310 µm
J Moon [14]	23(IVB)/18(IVB + IVTA)	IVB: bevacizumab 1.25 mg; IVB + IVTA: bevacizumab 1.25 mg + subtenon triamcinolone acetonide 40 mg	Primary outcomes: the number of additional IVB injections due to recurred ME during 6 months; Secondary outcomes: 1) changes in BCVA and CMT from baseline to 6 months; 2) BCVA, and CMT at 1, 3 and 6 months after injection; 3) time point of additional IVB injection; 4) safety profifiles including adverse effects such as IOP elevation, retinal detachment, endophthalmitis, and vitreous hemorrhage were evaluated	Mon 1, 3, 6	1) logMAR visual acuity ≥ 0.3 (Snellen equivalent ≤ 20/40); 2) ME secondary to BRVO
Chuanfeng Fan [15]	30(IVR)/27(IVR + IVTA)	IVR: ranibizumab 0.5 mg; IVR + IVTA: ranibizumab 0.5 mg + triamcinolone acetonide 1 mg	1) CMT; 2) intraocular pressure(IOP); 3) BCVA	Mon 1, 2, 3, 4, 5, 6	1) symptom duration < 3 months; 2) best-corrected visual acuity (BCVA) worse than 20/40 (early treatment of diabetic retinopathy study [ETDRS] equivalent = 70 letters); 3) central macular thickness (CMT) ≥ 275 μm; 4) fundus fluorescein angiogram (FFA) showing wide-spread diffuse leakage involving the macular and the fovea
Peter A. Campochiaro [16]	23(IVA); 23(IVTA + IVA)	IVA: triamcinolone acetonide(Suprachoroidal Injection) plus aflibercept IVTA + IVA: plus aflibercept	Primary outcomes: the number of protocol-required aflflibercept re-treatments through month 3; Secondary outcomes: 1) mean improvement from baseline BCVA and CST at months 1, 2, and 3; 2) percentage of participants with CST ≤ 310 um at months 1, 2, and 3; 3) percentage of participants with BCVA gain ≥ 0, 5, 10 or 15 letters at months 1, 2, and 3; 4) percentage of participants with BCVA loss < 15 letters at months 1, 2, and 3	Mon 1, 2, 3	1) ≥ 18 years of age; 2) macular edema due to RVO for 12 months; 3) BCVA ≥ 20 in each eye (20/400 Snellen equivalent) and ≤ 70 in the study eye (20/40 Snellen equivalent); 4) Central subfield thickness (CST) was ≥ 310 μm
Kyungmin Lee [17]	31(IVTA)&95(IVB)/25(IVTA + IVB)	IVTA: triamcinolone acetonide 4 mg(0.1 ml); IVB: bevacizumab 2.5 mg(0.1 ml); IVTA + IVB: triamcinolone 2 mg(0.05 ml) + bevacizumab 1.25 mg(0.05 ml)	1) Visual acuity; 2) change in visual acuity; 3) intraocular pressure	Mon 1, 3, 6, 12, 24	1) Best corrected visual acuity in Snellen chart ≤ 20 / 40, or ≥ 20 / 400; 2) Center-involved macular edema secondary to BRVO present on clinical examination; 3) Media clarity; 4) papillary dilation; 5) subject cooperation sufficient for adequate fundus photographs
Raj K Maturi [18]	14(IVB)/11(IVB + DEX)	IVB: IVB 1.25 mg + sham DEX injection after 1 week IVB + DEX: 1.25 mg + DEX injection after 1 week;	Primary outcomes: improvement in VA at 6 months; Secondary outcomes: 1) the mean changes in CST; 2) the proportions of eyes with CST 250 μm; 3) the number of bevacizumab injections required by each group	Mon 6	1) macular edema of less than 1 year’s duration due to BRVO or CRVO; 2) central subfield thickness (CST) 250 μm; 3) Best corrected VA scores at baseline were 24 and 80 Early Treatment Diabetic Retinopathy Study (ETDRS) letters
ZHAO Xue-zhang [20]	20(IVTA)/20(IVTA + IVC)	IVTA: triamcinolone acetonide 1 mg IVTA + IVC: triamcinolone acetonide 1 mg + conbercept 0.5 mg	1) BCVA; 2) CMT; 3) intraocular pressure(IOP)	Week 1, Month 1, 3	1) Confirmed by optical coherence tomography (OCT) and fluorescence fundus angiography (FFA); 2) All cases were monocular; 3) Course of disease within 3 months; 4) The age range was 30–75 years, and the intraocular pressure was normal; 5) Relevant information is complete
DUAN Yu-ping [21]	150(IVTA)/150(IVTA + IVC)	IVTA: triamcinolone acetonide 0.05 ml IVTA + IVC: triamcinolone acetonide 0.05 ml + conbercept 0.05 ml	1) BVCA; 2) CMT; 3) intraocular pressure(IOP)	Week 1; Mon 1, 3, 6	1) RVO was confirmed by ophthalmic examination; 2) Chief complaint of vision deformity or vision loss; 3) Fundus examination showed superficial retinal hemorrhage in the affected vein area, accompanied by digital redness of the retina, detour and expansion of blocked veins, accompanied by white sheath; 4) Fundus fluorescein angiography (FFA) showed delayed filling time of retinal vein accompanied by vascular wall leakage; 5) Optical coherence tomography (OCT) revealed the elevation or disappearance of macular fovea, diffuse thickening of retina, and macular fovea thickness (CMT) > 250 μm; 6) The right eye was selected for binocular involvement, and the unilateral eye was selected for unilateral involvement; 7) The intraocular pressure of the affected eye was normal; 8) Obtaining informed consent of patients and their families; 9) Approved by the hospital ethics Committee
Zheng Tan [19]	33(IVA)/33(IVA + IVTA)	IVA: aflibercept 0.05 ml, monthly follow-up after 3 injections, repeated injection if necessary (3 + Pm) IVA + IVTA: aflibercept 0.05 ml was injected intravitreal, and 40 mg(0.5 ml) of triamcinolone acetonide was injected subfascia	1) BCVA; 2) CMT; 3) intraocular pressure(IOP)	Month 1, 3, 6	Patients diagnosed with macular edema secondary to CRVO

**Fig. 2:**
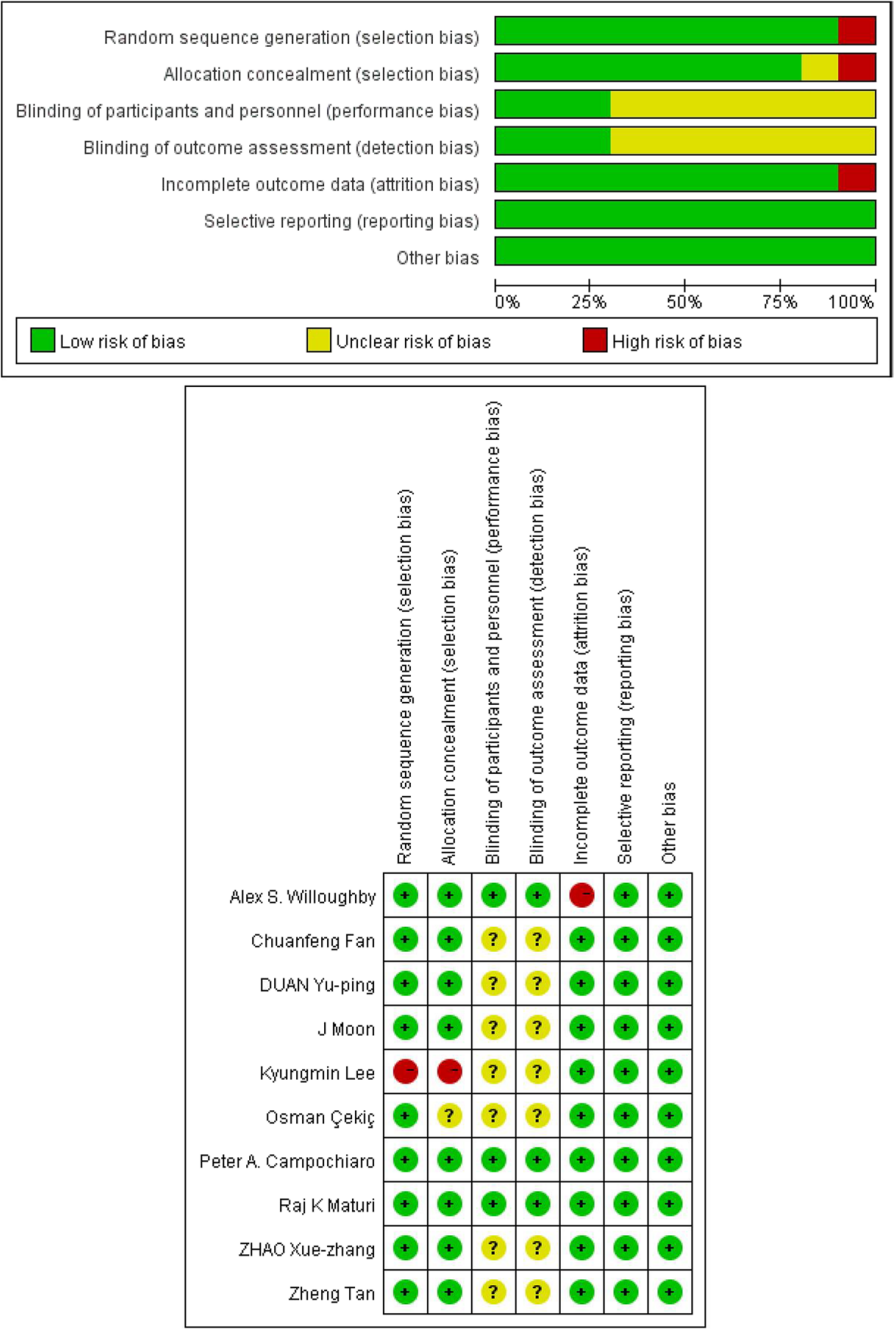
Risk of bias for all included studies

The risk of bias for a total of 9 RCTS [12–16, 18–21] were assessed on the basis of the Cochrane Collaboration tool (Fig. 2). Besides, we assessed the bias risk of non-RCT [17] according to MINORS, and concluded that the score of this non-RCT was 20, indicating ideal quality (Supplement Table 2).

### Best-corrected visual acuity

**Fig. 3:**
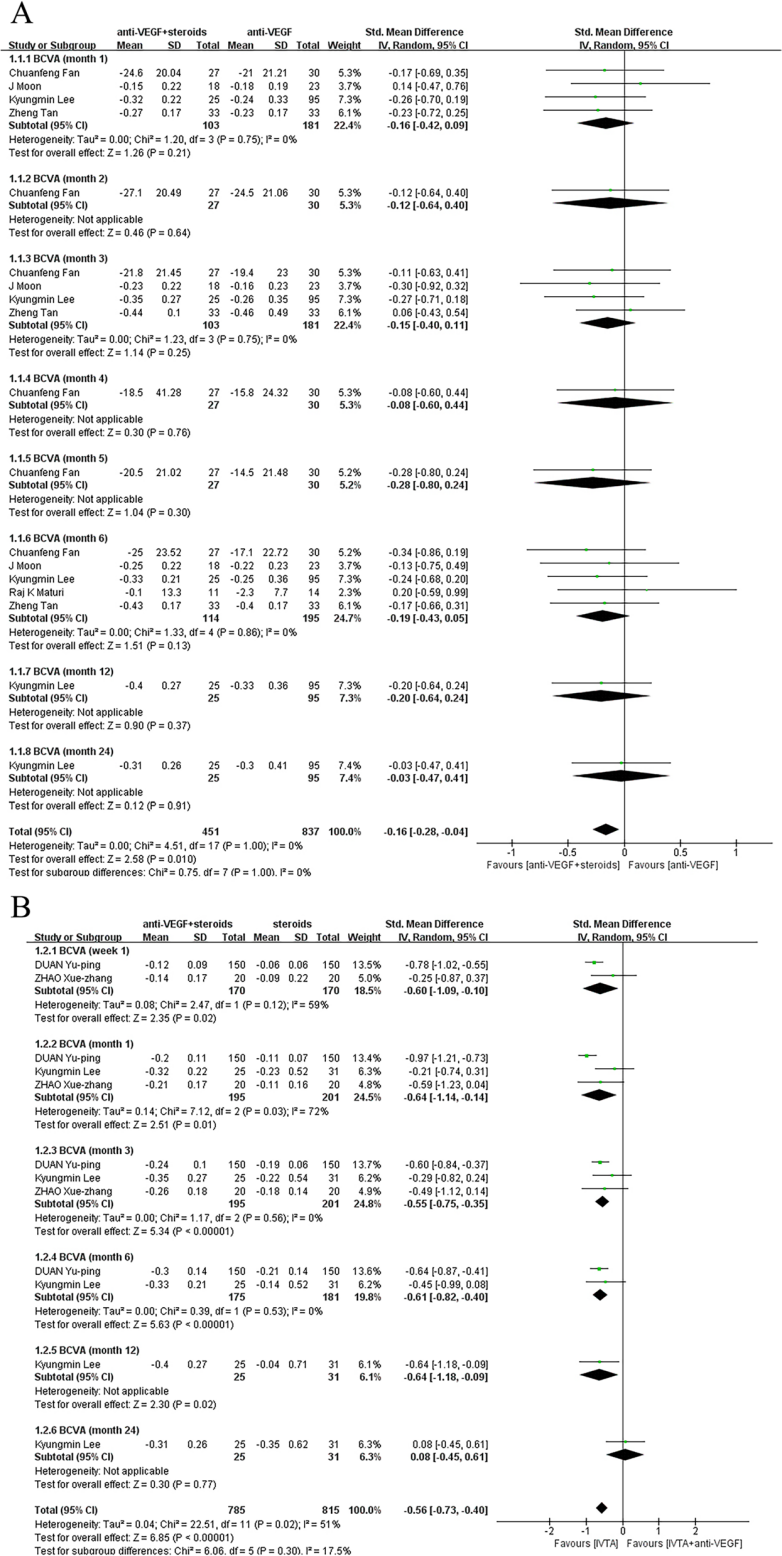
**A** Control group was treated with anti-VEGF monotherapy. Combined treatment was associated with a more significant improvement (pooled SMD, 95% CI, -0.16 [-0.28, -0.04], *P* = 0.01) in BCVA compared with control group. **B** Control group was treated with steroids monotherapy. There was a significant improvement (pooled SMD, 95% CI, 0.56 [-0.73, -0.40], *P* < 0.00001) in BCVA

Some trials reported measuring VA with Snellen diagrams and then converted the data to logMAR for analysis. In order to avoid misleading conversion of Snellen data to LogMAR, effect indicators of SMD were used in BCVA analysis to eliminate the influence of different measurement methods or different units. In order to observe the effect of follow-up duration on efficacy of treatment regimen, subgroups were established according to different time points. Whether the control group was treated with anti-VEGF monotherapy or steroids monotherapy, the effect of combination therapy on ME secondary to RVO was superior to that of monotherapy, remaining unacted on follow-up times of trails. Combined treatment was associated with a more significant improvement (pooled SMD, 95% CI, -0.16 [-0.28, -0.04], *P* = 0.01) in BCVA compared with anti-VEGF monotherapy in month 1 (SMD, 95% CI, -0.16 [-0.42, 0.09], *P* = 0.21), month 2 (SMD, 95% CI, -0.12 [-0.64, 0.40], *P* = 0.64), month 3(SMD, 95% CI, -0.15 [-0.40, 0.11], *P* = 0.75), month 4(SMD, 95% CI, -0.08 [-0.60, 0.44], *P* = 0.76), month 5 (SMD, 95% CI, -0.28 [-0.80, 0.24], *P* = 0.30), month 6 (SMD, 95% CI, -0.19 [-0.43, 0.05], *P* = 0.13), month 12 (SMD, 95% CI, -0.20 [-0.64, 0.24], *P* = 0.37), month 24 (SMD, 95% CI, -0.20 [-0.64, 0.24], -0.03 [-0.47, 0.41], *P* = 0.91) (Fig. 3a). Combined to steroids monotherapy, combination treatment also led to a more significant improvement (pooled SMD, 95% CI, -0.56 [-0.73, -0.40], *P* < 0.00001) in BCVA in week 1 (SMD, 95% CI, -0.60 [-1.09, -0.10], *P* = 0.02), month 1 (SMD, 95% CI, -0.64 [-1.14, -0.14], *P* = 0.01), month 3 (SMD, 95% CI, -0.55 [-0.75, -0.35], *P* < 0.00001), month 6 (SMD, 95% CI, -0.61 [-0.82, -0.40], *P* < 0.00001), month 12 (SMD, 95% CI, -0.64 [-1.18, -0.09], *P* = 0.02), month 24 (SMD, 95% CI, 0.08 [-0.45, 0.61], *P* = 0.77) (Fig. 3b).

### Central choroidal thickness

Both the retina and choroid are rich in blood vessels, and studies have shown that choroid blood flow and thickness affect the outcomes of RVO. It has been reported that the recurrence of ME is low in cases of choroid thinning [22]. It has also been reported that the thickening of the retina and choroid indicates the severity of the obstructive lesions in RVO, but it also means that the improvement does not mean the resumption of blood flow circulation within the vessels of the obstructed retina [23]. Changes in CMT, VCT (vascular choroidal thicknes), SCT (stromal choroidal thickness), TCT (total choroidal thickness), SCS (suprachoroidal space thickness), are therapeutic indicators to evaluate choroid thickness.

**Fig. 4:**
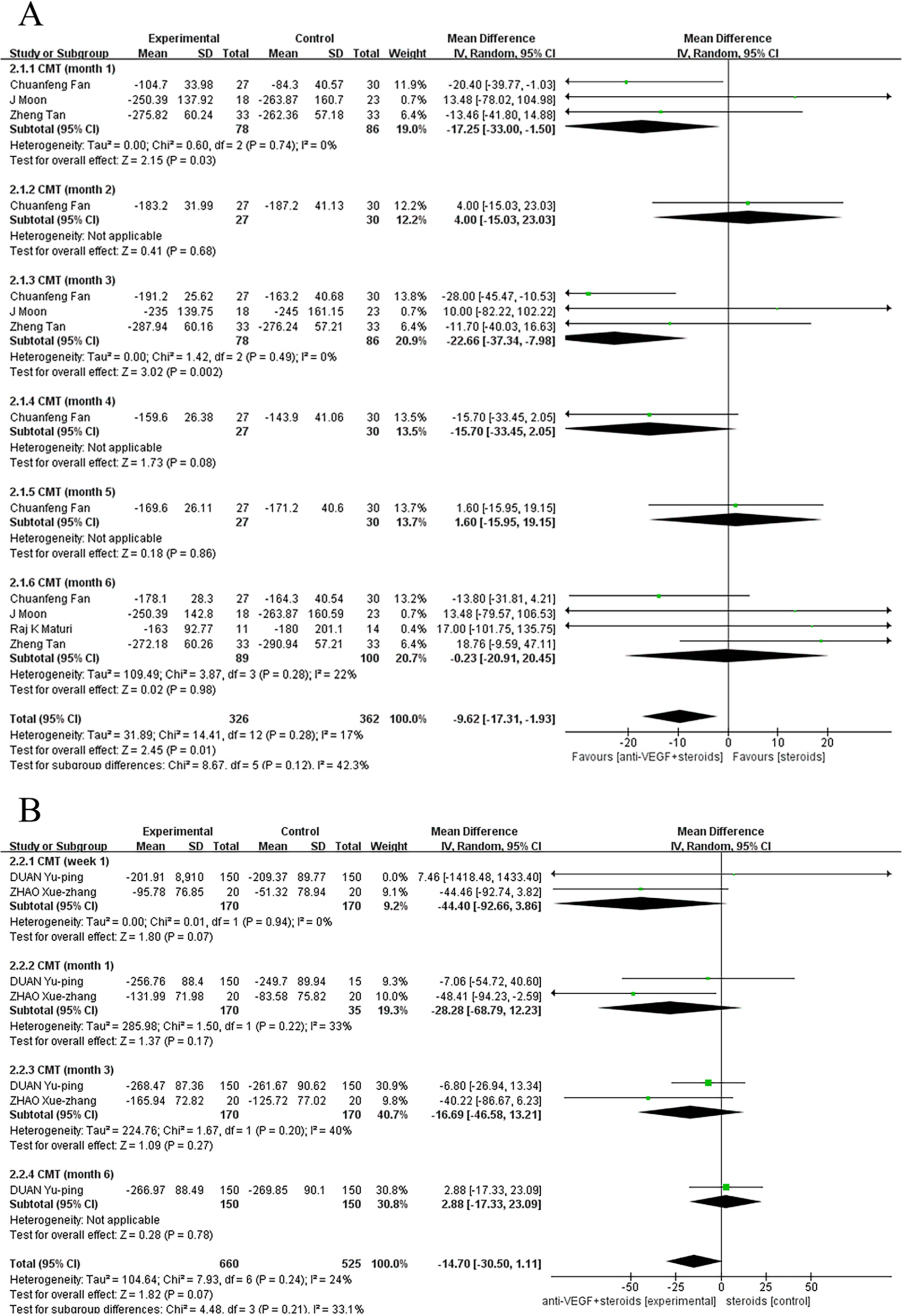
**A** Control group was treated with anti-VEGF monotherapy. Compared with monotherapy group, the combination therapy had a better effectiveness at improving on CMT (pooled MD, 95%CI, -9.62 [-17.31, -1.93], *P* = 0.01). **B** Control group was treated with steroids monotherapy. Data showed no significant difference in efficacy between monotherapy and combination therapy at improving on CMT

When the control group was treated with anti-VEGF monotherapy, there was a statistically significant difference between the two treatments. Compared with monotherapy group, the combination therapy had a better effectiveness (pooled MD, 95% CI, -9.62 [-17.31, -1.93], *P* = 0.01) at improving on CMT in month 1(MD, 95% CI, -17.25 [-33.00, -1.50], *P* = 0.03), month 2 (MD, 95% CI, 4.00 [-15.03, 23.03], *P* = 0.68), month 3 (MD, 95% CI, -22.66 [-37.34, -7.98], *P* = 0.002), month 4 (MD, 95% CI, -15.70 [-33.45, 2.05], *P* = 0.08), month 5 (MD, 95% CI, 1.60 [-15.95, 19.15], *P* = 0.86), month 6 (MD, 95% CI, -0.23 [-20.91, 20.45], *P* = 0.98) (Fig. 4a). However, when the control group was treated with steroids monotherapy, pooling the data of these studies showed no significant difference in efficacy between monotherapy and combination therapy, regardless of the duration of the follow-up (Fig. 4b).

**Fig. 5:**
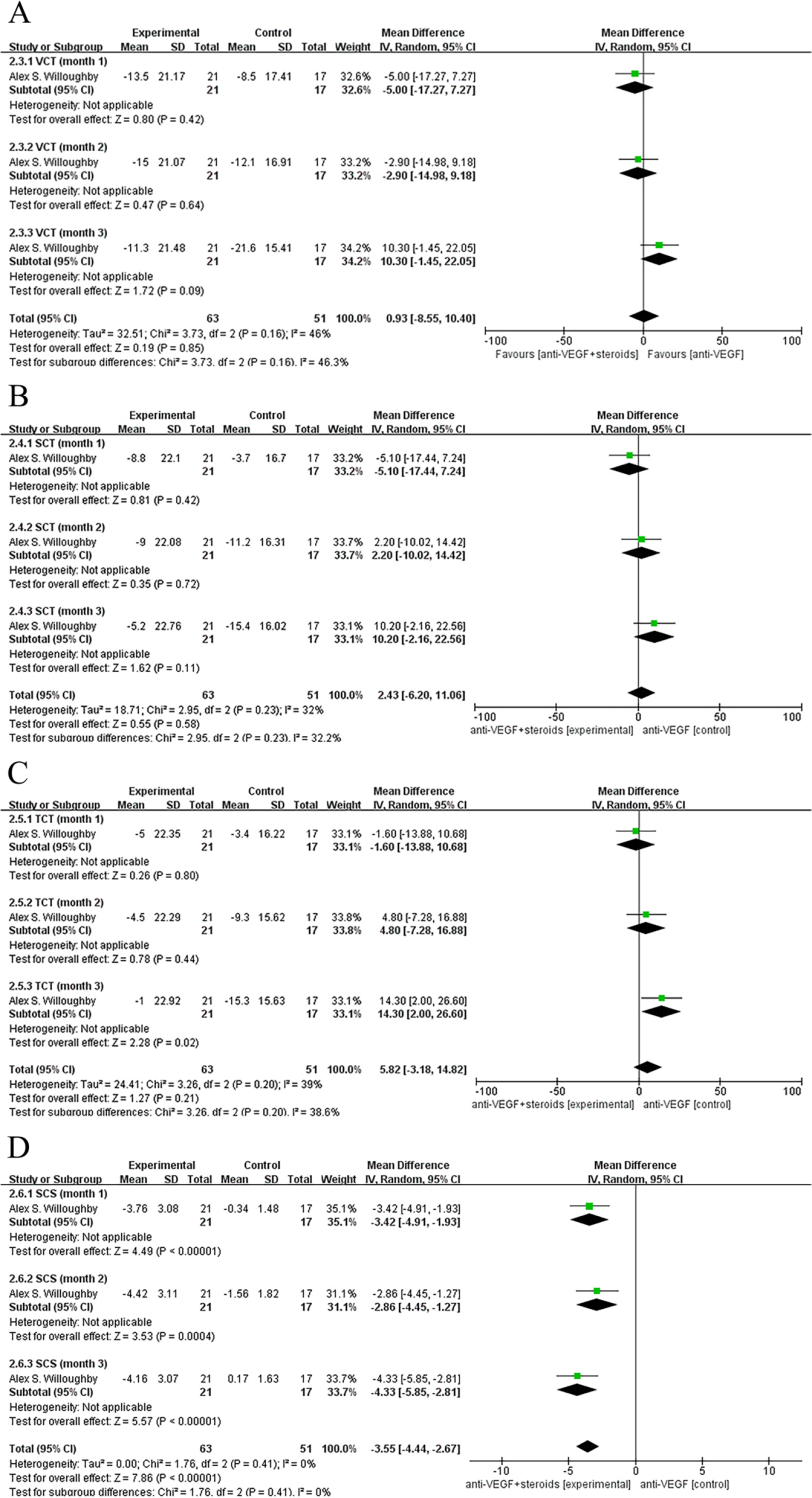
Control group was treated with anti-VEGF monotherapy. **A**, **B**, **C** There was no significant difference between the two treatments. **D** A significantly higher improvement in SCS was found in the combination therapy group compared with control group on follow-up (pooled MD, 95% CI, -3.55 [-4.44, -2.67], *P* < 0.00001)

Only one study [13] measured other related indicators (VCT, SCT, TCT,SCS) of central choroid thickness, and there was no significant difference between the two treatments when the control group was treated with anti-VEGF monotherapy, except that a significantly higher improvement in SCS was found in the combination therapy group compared with the anti-VEGF monotherapy group on follow-up (pooled MD, 95% CI, -3.55 [-4.44, -2.67], *P* < 0.00001) (Fig. 5a, b, c, d).

### Intraocular pressure

**Fig. 6:**
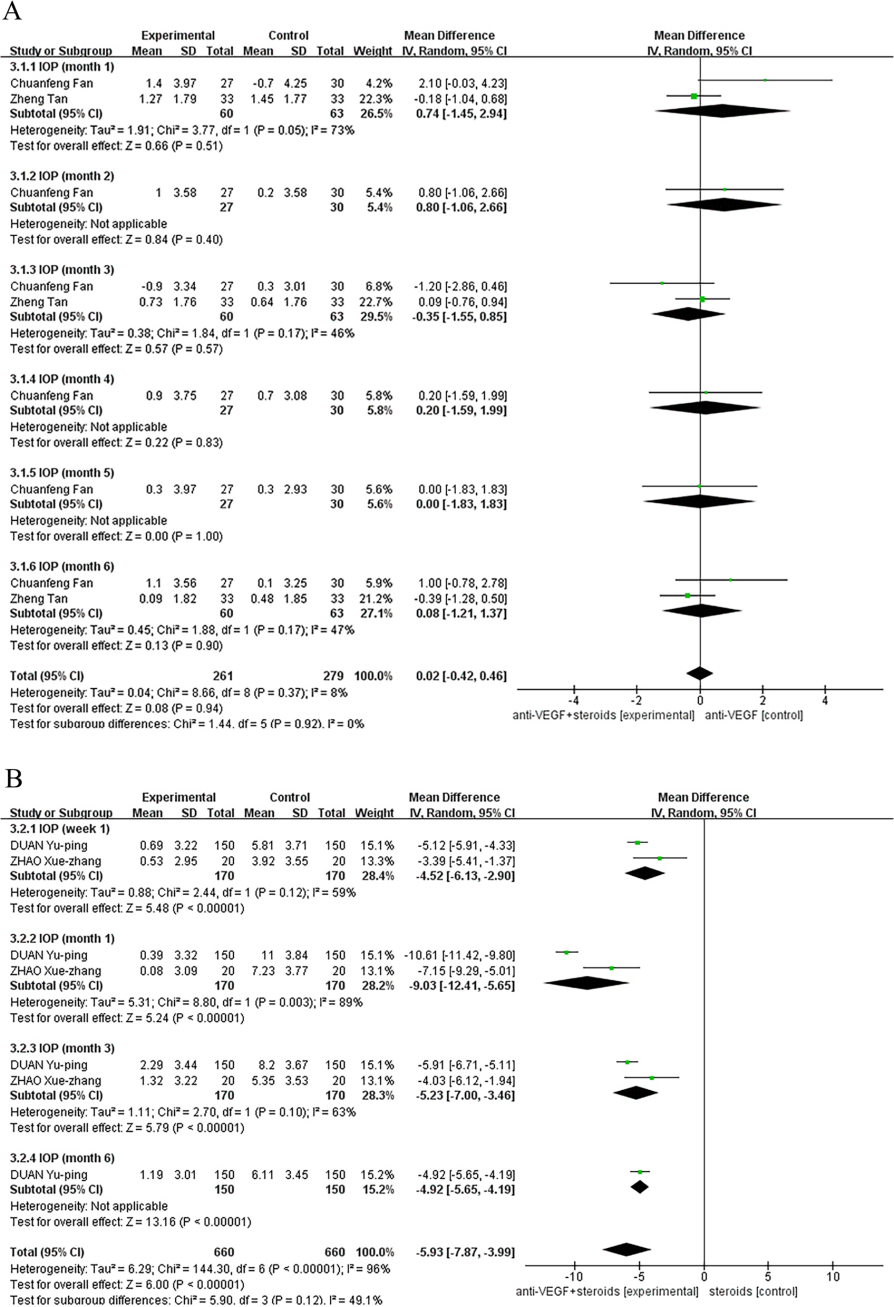
**A** Control group was treated with anti-VEGF therapy. No significant difference was found in the effect of two treatments on changes in IOP compared with baseline between the combination group and control groups. **B** Control group was treated with steroids monotherapy. Combination therapy was associated with a better relief of IOP with a significantly difference compared to control group on follow-up (pooled MD, 95% CI, -5.93 [-7.87, -3.99], *P* < 0.00001)

When the control group received anti-VEGF therapy, no significant difference was found in the effect of two treatments on changes in IOP campared with baseline between the experimental and control groups (Fig. 6a). Combination therapy was associated with a better relief of IOP with a significantly difference compared to steroids monotherapy group on follow-up (pooled MD, 95% CI, -5.93 [-7.87, -3.99],*P* < 0.00001). The results were consistent at all follow-up time points of week 1 (MD, 95% CI, -4.52 [-6.13, -2.90] *P* < 0.00001), month 1 (MD, 95% CI, -9.03 [-12.41, -5.65], *P* < 0.00001), month 3 (MD, 95% CI, -5.23 [-7.00, -3.46], *P* < 0.00001), or month 6 (MD, 95% CI, -4.92 [-5.65, -4.19], *P* < 0.00001) (Fig. 6b).

### Incidence of adverse events

Studies included in this meta-analysis reported some of the complications that had a zero incidence in both groups, including endophthalmitis, retinal detachment, vitreous hemorrhage. Moreover, some occasional adverse events, such as macular fibrosis and anterior chamber inflammation, were not included in the analysis, for only one trial had reported these complications existing in monotherapy group. Cataract, intraocular hypertension and visual acuity reduced were included in the analysis for incidence of adverse events.

**Fig. 7:**
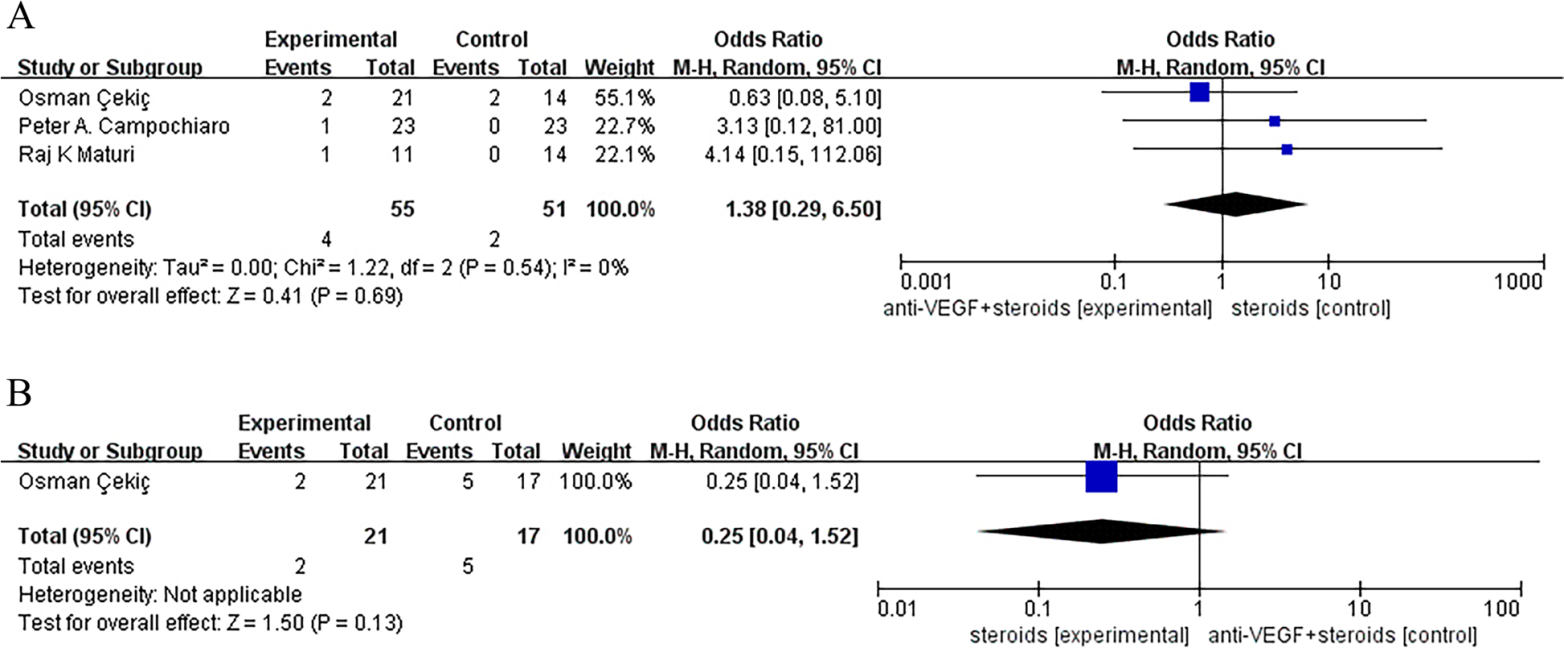
**A**, **B** No statistical significant difference was found between monotherapy and combination therapy groups in the incidence of cataract

**Fig. 8:**
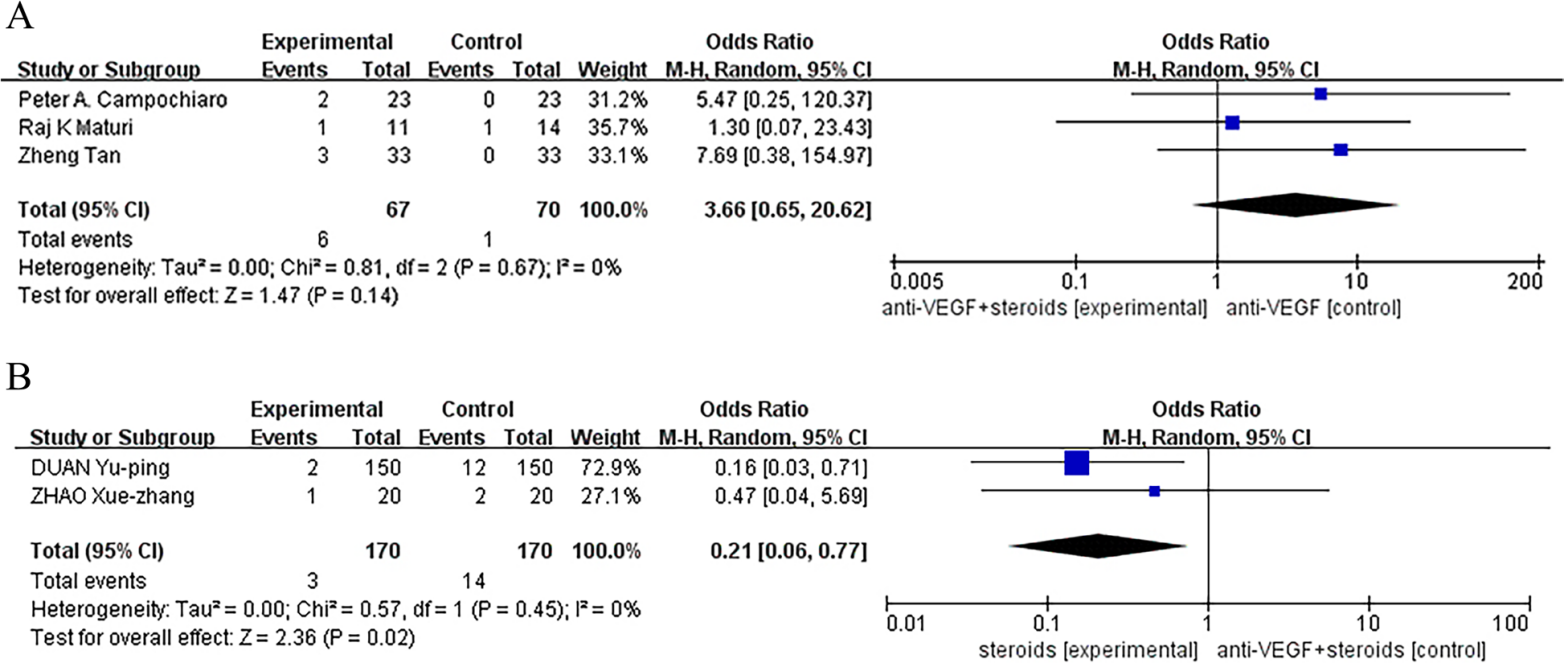
**A** Control group was treated with anti-VEGF monotherapy. No statistical significant differences was found between monotherapy and combination therapy groups in the incidence of ocular hypertension. **B** Control group was treated with steroids monotherapy. There was a statistical significant difference was found between monotherapy and combination therapy groups in the incidence of ocular hypertension (Odds Ratio, 95% CI, 0.21 [0.06, 0.77], *P* = 0.02)

**Fig. 9:**
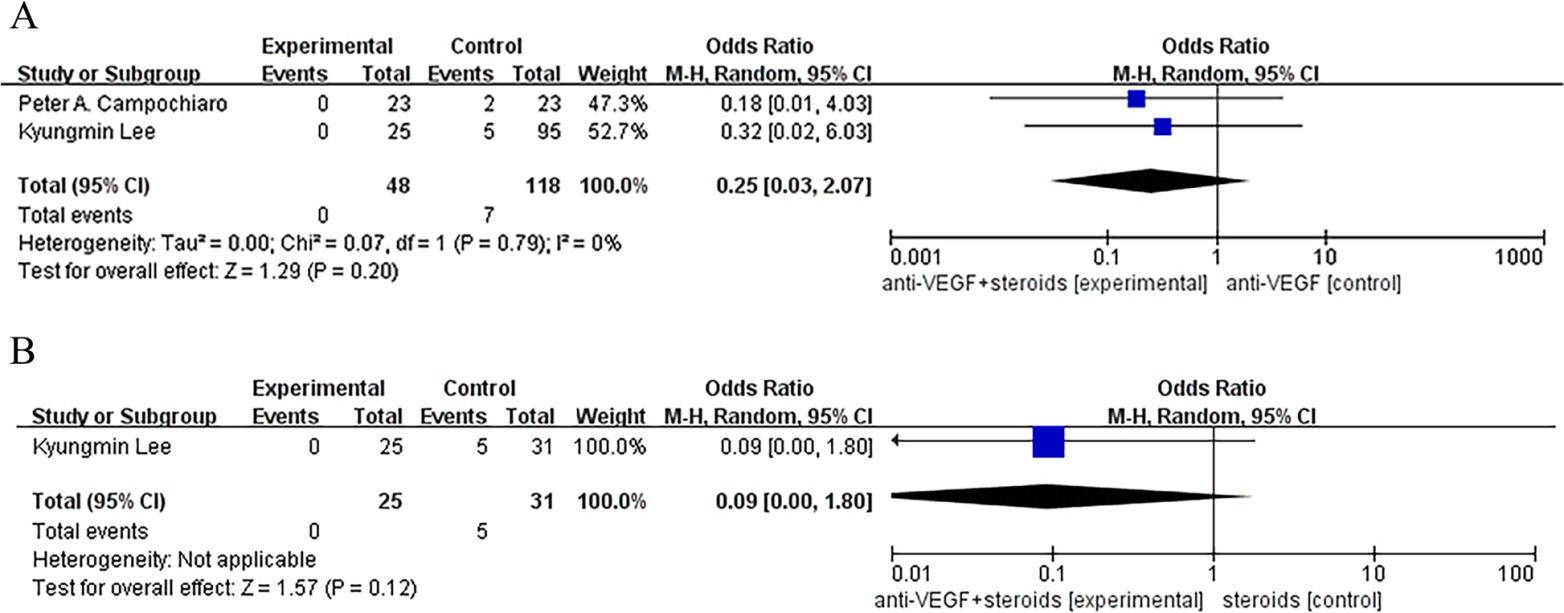
**A**, **B** No statistical significant difference was found between monotherapy and combination therapy groups in the incidence of visual acuity reduced

We did not find a statistical significant difference between monotherapy and combination therapy groups in the incidence of cataract, ocular hypertension and visual acuity reduced, except that a less common incidence of ocular hypertension was found in combined group compared with control group receiving steroids (Odds Ratio, 95% CI, 0.21 [0.06, 0.77], *P* = 0.02) (Figs. 7, 8 and 9).

### Average time to first anti-VEGF reinjection

**Fig. 10:**
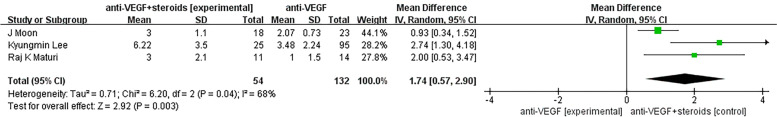
Control group was treated with anti-VEGF monotherapy. Data showed that the combination group was associated with a longer average time to first anti-VEGF reinjection (MD, 95% CI, 1.74 [0.57, 2.90], *P* = 0.003)

During follow-up, intravitreal reinjections were performed with initial anti-VEGF if fundus examination or optical coherence tomography (OCT) showed recurrent ME associated with decreased visual acuity. Compared to control group treated with anti-VEGF alone, the combination group was associated with a longer average time to first anti-VEGF reinjection (MD, 95% CI, 1.74 [0.57, 2.90], *P* = 0.003) (Fig. 10). The results favored the conclusion that combination therapy could shorten the terms between treatments.

## Discussion

Our results of analysis showed that patients receiving the combination of anti-VEGF and steroids therapy were more likely to achieve the target of improving BCVA compared with patients receiving anti-VEGF or steroids therapy alone. Compared to anti-VEGF therapy alone, the combination of anti-VEGF and steroids therapy offers a greater reduction of CMT and SCS, and this combination can significantly reduce IOP compared to steroids therapy alone, thereby reducing ocular hypertension complications after treatment.

Combination therapy of steroids with anti-VEGF has a better safety profile than steroids therapy alone, a longer duration of therapeutic effect than anti-VEGF therapy alone and a better cost effectiveness than DEX implants. Besides, the greatest advantage of combination therapy is that once the number of injections is reduced, the time from the first injection to the next injection is longer than that of monotherapy. Compared to traditional monotherapy with a high injection frequency, the convenience of treatment and lack of identifiable complications for patients can relieve the pressure of receiving therapy and improve the quality of life of patients. What’s more, patients receiving combined treatment required fewer anti-VEGF reinjections compared to those receiving monotherapy, according to data from several trials [15, 16, 18]. Chuanfeng Fan [15] reported that the average number of injections was 4.23 ± 0.56 in the IVR (intravitreal ranibizumab), which was higher than the 3.42 ± 0.41 injections given to the IVR + IVTA group. Peter A. Campochiaro [16] reported that the average number of injections was 23 in IVA (intravitreal aflibercept) group, higher than 9 in IVA + IVTA group. Raj K Maturi [18] reported that the median number of IVB (intravitreal bevacizumab) injections was 2 in the IVB + DEX group and 3 in the IVB group. In shorten, combination therapy can reduce the average number of injections given to patients.

Studies have found that retinal pigment epithelial cells, as an important component of the blood-retina barrier, which play an important role in regulating ocular inflammation, are able to interact with leukocytes extensively infiltrating in the choroidal circulation and respond to IL-1β and TNF-α by secreting chemokines including IL-8 and MCP-1, while ICAM-1 mediates VEGF-induced retinal vascular permeability. DEX can inhibit IL-1β induction of MCP-1 and IL-8 by human retinal pigment epithelial cells (HRPE) [24], while TA inhibits the interaction between leukocytes and endothelial cells by reducing the expression of P-selectin and ICAM-1, thereby alleviating ME [25]. Steroids inhibit the production of various inflammatory cytokines that promote leukocyte adhesion and the destruction of blood-retinal barrier, thus treating macular edema, which may explain why the number of injections can be reduced [26]. IOP is one of the expected side effects of intraocular corticosteroid treatment [27]. The less frequent injections may be attributable to a lower rate of IOP in the combination therapy group.

Taken together, our results were robust and consistent, independent of the length of follow-up, indicating that anti-VEGF with steroids combination therapy can be used as a therapeutic strategy to improve ME secondary to RVO, showing a likehood of solving the problem that laser photocoagulation is ineffective for ME secondary to CRVO. Combination therapy is more cost-effective and can reduce the dose and injection frequency to some extent, lower the risk of complications and achieve better outcomes of visual acuity compared with monotherapy.

The limitation of our study is that the number of samples that can be included in the study is small for analysis of each outcome indicator. For example, it showed no significant difference in the efficacy of two treatments on VCT, SCT and TCT, likely to be a result of only one trial included in this analysis group. Endophthalmitis is probably the most visually destructive complication of intraocular injection, despite its low incidence [28]. More trials and a larger number of patients may contribute to more reliable results of complication incidence. Additionally, included trials selected different anti-VEGF drugs such as ranibizumab or aflibercept, and different steroids such as triamcinolone acetonide or dexamethasone implant, while the types or manufacturers of the drugs may also affect the efficacy. Diverse methods for injecting such as traditional intraocular injection or suprachoroidal Injection, may also offer a difference in effectiveness.

In this study, the analysis was not performed according to the detailed classification of RVO. Based on the anatomical location of the occlusion, RVO can be divided into CRVO and BRVO, and BRVO can be further divided into major BRVO and macular BRVO [29]. Previous studies have confirmed that the levels of inflammatory cytokines and VEGF in the aqueous humor of major BRVO are significantly higher compared to macular BRVO, so major BRVO requires more and longer anti-VEGF treatments [30]. In addition, CRVO can be clinically divided into ischemic-CRVO (I-CRVO) and non-ischemic CRVO (NI-CRVO). In NI-CRVO patients, the severity of macular edema is significantly correlated with macular retinal epithelial pigment degeneration and serous macular detachment. In terms of resolution of macular edema, visual improvement was better in NI-CRVO compared with I-CRVO [31]. Therefore, the response of RVO to different treatment regimens may be associated with the classification of RVO.

Although further trials and studies needs to be conducted to establish the optimal range of application in detail of anti-VEGF with steroids combination therapy in the clinical treatment of ME secondary to RVO, evidence of our findings suggest that this combination is superior to monotherapy in improving clinical indicators and reducing adverse events, especially refractory and recurrent ME secondary to RVO.

## Supplementary Information

**Additional file 1:** **Supplement Figure 1.** The publication bias of the effect of anti-VEGF monotherapy and vs combined therapy on BCVA at 6-month follow-up.

**Additional file 2:** **Supplement Table 1.** The publication bias of the effect of anti-VEGF monotherapy and vs combined therapy on BCVA at 6-month follow-up. The *P* value was less than 0.05, indicating that no significant publication bias was detected.

**Additional file 3: Supplement Table 2.** Bias risk of non-RCT according to MINORS.

## Data Availability

The data used in our study can be found in the corresponding published studies, as the references shown in the manuscript. The datasets used and/or analysed during the current study are available from the corresponding author on reasonable request.

## Abbreviations

RVO: Retinal vein occlusion
BRVO: Branch retinal vein occlusion
ME: Macular edema
CRVO: Central retinal vein occlusion
VEGF: Vascular endothelial growth factor
CNKI: China National Knowledge Infrastructure
RCTs: Random clinical trials
BCVA: Best-corrected visual acuity
CMT: Central macular thickness
IOP: Intraocular pressure
SMD: Standardized mean difference
MD: Mean difference
VEGF-R: VEGF receptor (VEGF-R)
EURETINA: The European Society of Retina Specialists
TA: Triamcinolone acetonide
DEX: Dexamethasone vitreous implant
IVTA: Injection of triamcinolone acetonide
Ozurdex: Dexamethasone sustained-release implant
SD: Standard deviation
MINORS: Methodological Index for Non-randomized Studies
CI: Confidence interval
VCT: Vascular choroidal thicknes
SCT: Stromal choroidal thickness
TCT: Total choroidal thickness
SCS: Suprachoroidal space thickness
OCT: Optical coherence tomography
IVR: Intravitreal ranibizumab
IVB: Intravitreal bevacizumab
HRPE: Human retinal pigment epithelial cells
I-CRVO: Ischemic-CRVO
NI-CRVO: Non-ischemic CRVO
