# Supplementary figures and images for: Efficacy and effectiveness of anti-VEGF or steroids monotherapy versus combination treatment for macular edema secondary to retinal vein occlusion: a systematic review and meta-analysis
Source: BMC Ophthalmol. 2022 Dec 6;22:472. doi: 10.1186/s12886-022-02682-7 (PMC9727869; doi:10.1186/s12886-022-02682-7)

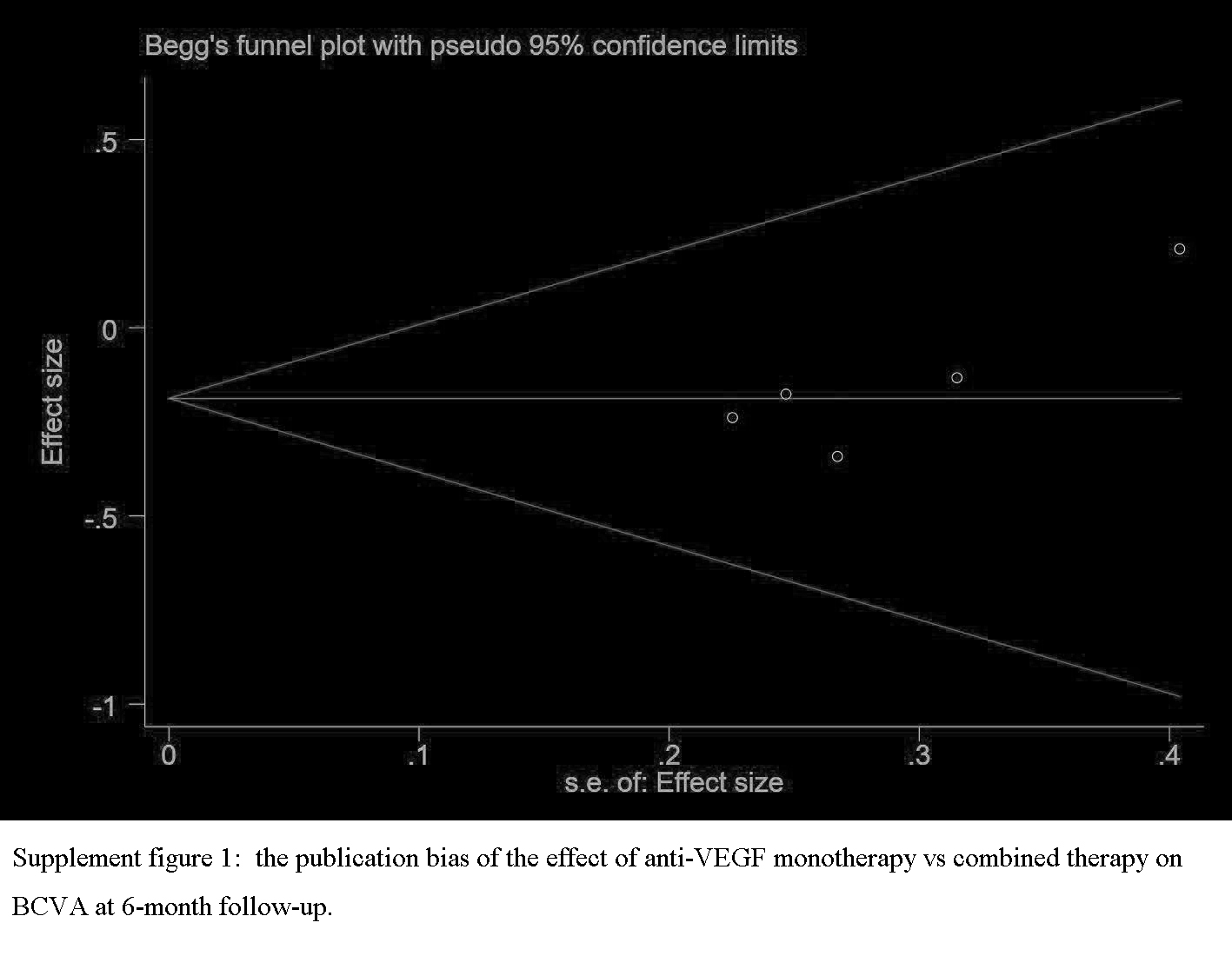

Supplement: Supplementary file 1 — Additional file 1: Supplement Figure 1. The publication bias of the effect of anti-VEGF monotherapy and vs combined therapy on BCVA at 6-month follow-up. [file 12886_2022_2682_MOESM1_ESM.jpg]

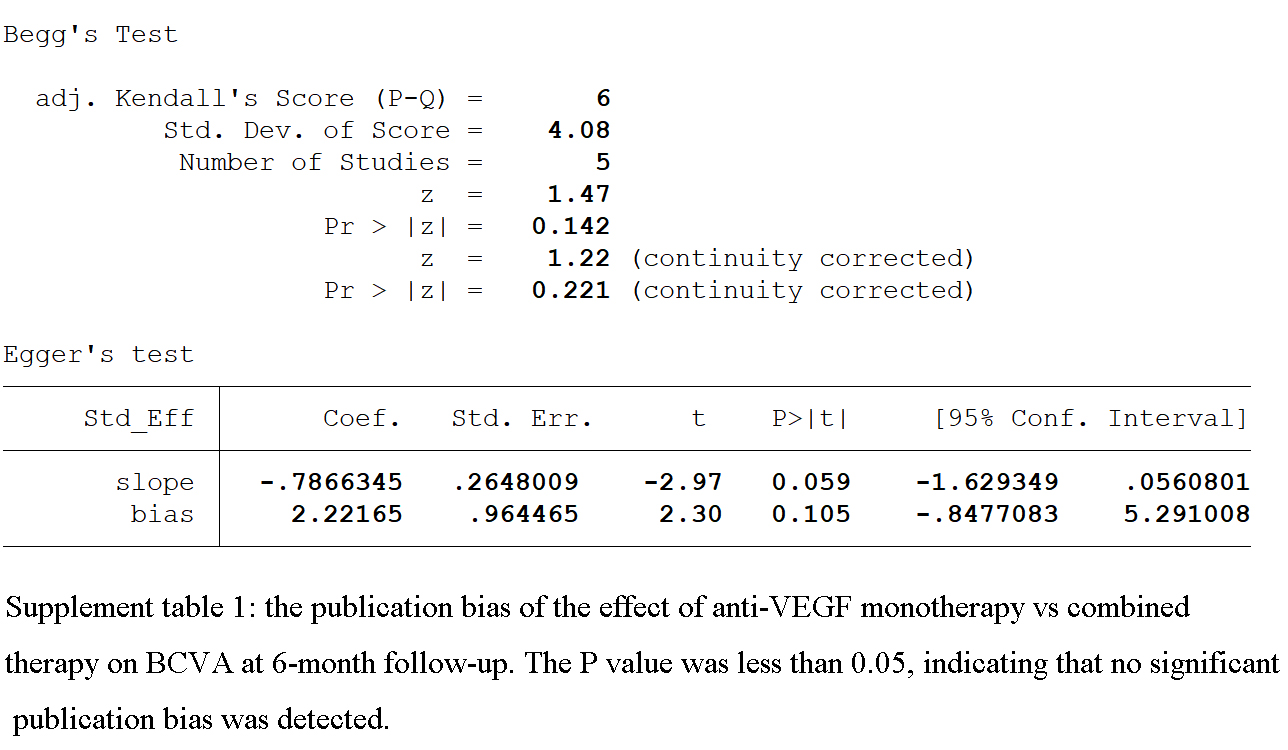

Supplement: Supplementary file 2 — Additional file 2: Supplement Table 1. The publication bias of the effect of anti-VEGF monotherapy and vs combined therapy on BCVA at 6-month follow-up. The P value was less than 0.05, indicating that no significant publication bias was detected. [file 12886_2022_2682_MOESM2_ESM.jpg]

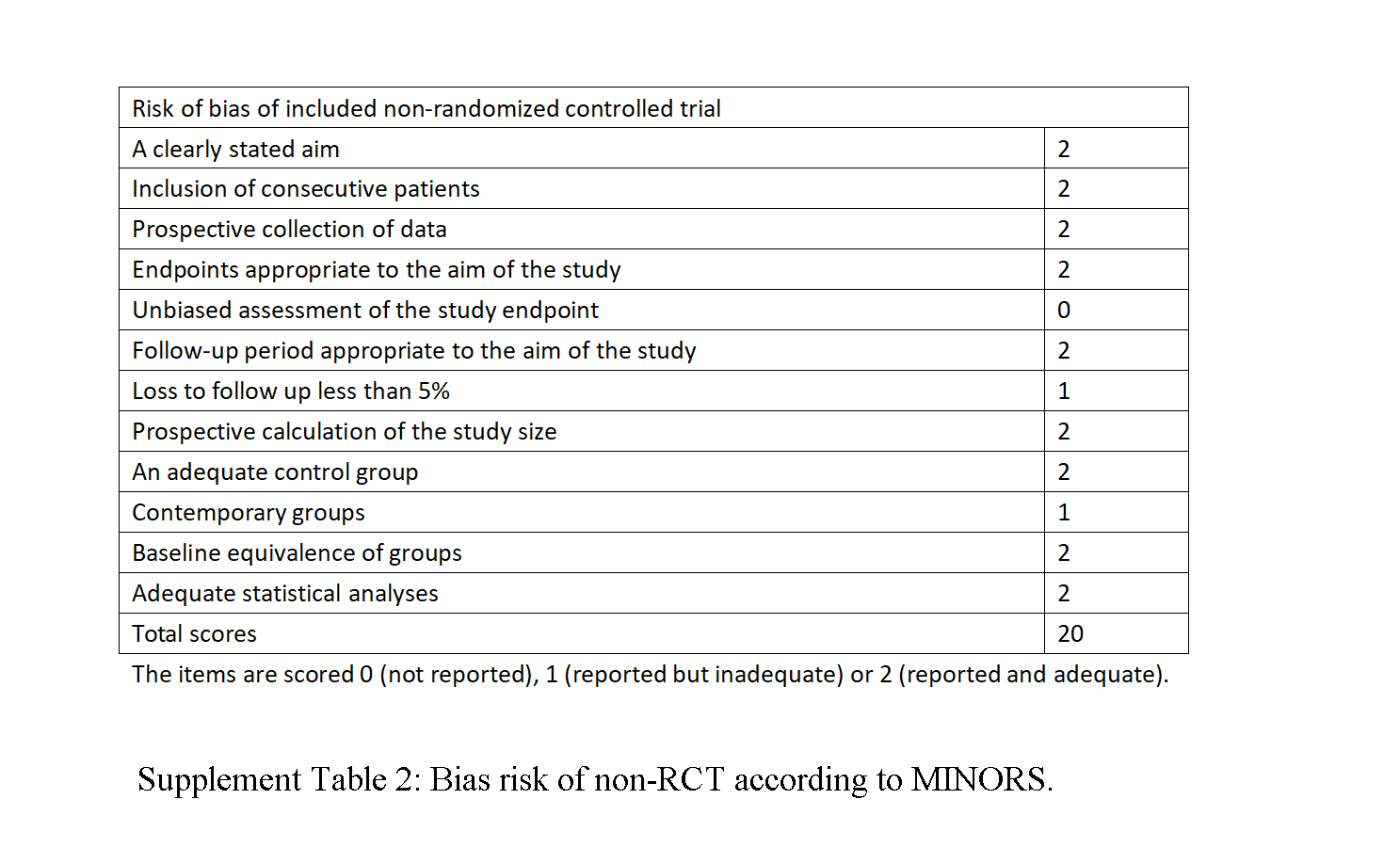

Supplement: Supplementary file 3 — Additional file 3: Supplement Table 2. Bias risk of non-RCT according to MINORS. [file 12886_2022_2682_MOESM3_ESM.jpg]
